# Raising capital amid economic policy uncertainty: an empirical investigation

**DOI:** 10.1186/s40854-022-00379-w

**Published:** 2022-08-15

**Authors:** Dawood Ashraf, Mohsin Khawaja, M. Ishaq Bhatti

**Affiliations:** 1 Islamic Development Bank Institute (A Member of Islamic Development Bank Group), 8111 King Khalid Street, Jeddah, 22332-2444 Saudi Arabia; 2 School of Business Studies, Institute of Business Administration (IBA), Karachi, Pakistan; 3 S P Jain School of Global Management, Sydney, NSW 2141 Australia; 4 La Trobe Business School, La Trobe University, Melbourne, Australia

**Keywords:** Economic policy uncertainty, Political uncertainty, Capital issuance, Debt and equity markets, Ownership structure, Governance mechanisms, C54, D81, E41, G32, G34, P16

## Abstract

This paper investigates how economic policy uncertainty affects firms’ frequency and their choice of financial instruments to raise capital. By applying a three-step sequential framework over a sample of 6834 publicly listed US non-financial firms, we find that during periods of high economic uncertainty, firms raise capital more frequently with a preference toward debt financing. The empirical findings suggest that firms prefer debt financing over equity financing to avoid ownership dilution and high equity premia. The rise in leverage during periods of high economic uncertainty highlights the importance of scrutinizing policy tools used to stabilize the economy during such times.

“*…this year [2020] the world’s non-financial firms have raised an eye-popping $3.6trn in capital from public investors*.^1^” (The Economist)

## Introduction

Over the past two decades, firms have operated in an increasingly uncertain economic environment, and financial markets have experienced significant volatility. Consequences of uncertainty include a decline in business activity (Işık et al. 2020), rising financing costs (Waisman et al. 2015), wider yield spreads (Bradley et al. 2016), shorter maturities of debt financing (Datta et al. 2019), and elevated risk premia for equity investments (Li 2017; Pástor and Veronesi 2013). We posit that when firms operate in an uncertain economic environment, there is an increased demand for capital to mitigate adverse effects brought by the macroeconomic environment.

Corporate finance literature offers several explanations about firms’ decision to raise capital and their choice of financing instruments (Haddad and Lotfaliei 2019; Nagar et al. 2019; Baker and Wurgler 2002; Myers and Majluf 1984; Jensen and Meckling 1976; Modigliani and Miller 1958; Ross 1977).^2^ However, the intervention of central banks during economic crises^3^ through quantitative easing and asset purchase programs and shifts in ownership structure of US firms^4^ has altered funding mechanisms and influenced risk-averse firms’ choice of financial instrument toward relatively “safe” bonds and leverage (Giambona et al. 2020; Kurtzman and Zeke 2017).

Building on our initial postulation that firms raise more capital during periods of high economic uncertainty, we hypothesize that the ownership structure of firms plays a significant role in determining the choice of financial instrument to raise capital. As such, this paper addresses three interrelated questions. First, does economic uncertainty lead firms to raise capital more frequently? Second, how does a firm’s ownership structure affect the choice of financing from a range of instruments, namely bank loans, bonds, convertible bonds, preferred equity, and common equity? Finally, what determines the volume of issuance given the choice of financing instrument? We assert that the initial decision to raise capital, followed by a choice of security selection and financing volume, are sequential and reflect a firm’s policy choices.^5^ Addressing these questions concurrently requires the use of a simultaneous equation framework. The application of this framework is an important contribution of this study.

This study uses a sample of 45,635 firm-year records with 13,308 instances of capital financing for the period beginning January 1, 2000, until December 31, 2018.^6^ We find evidence suggesting a higher propensity for debt financing by a factor of 27.63% as the volume of debt per issuance, on average, exceeds that of equity during the sample period. We find a positive association between economic uncertainty and the decision of firms to raise capital, supporting evidence that the demand for capital is stimulated by economic uncertainty (Husted et al. 2019; Atta-Mensah 2004; Klein 1977; Hartman 1972). In line with pecking order theory, we find that high economic uncertainty is associated with an increased demand for capital and that debt-based securities are the instruments of choice.

We find support for the control hypothesis whereby shareholders, particularly in firms with a higher proportion of institutional investors, prefer to raise capital using debt-based instruments to avoid ownership dilution and higher equity premia (Admati et al. 2018; Badoer and James 2016; Ellul 2008; Levy 2019). This finding also complements Tran (2019) that higher economic uncertainty is associated with low corporate risk-taking.

By using interactive variables, we find that large firms raise lower volumes of capital in the presence of political uncertainty, indicating their risk-averse behavior (Chan et al. 1985). Further, we find that certain governance mechanisms play a significant role in the process of raising capital. As an alternative to the economic policy uncertainty index, we use the implied volatility index (VIX) as a measure of market uncertainty. The results remain robust despite using an alternative measure for economic uncertainty.

As a further robustness check, we adopt the multinomial logit model with sample selection (MLMSS) as an alternative methodology for empirical estimation. Unlike the sequential model with a categorical choice variable for financing instruments based on the pecking order theory, the choice decision variable under MLMSS does not consider a strict order. Essentially, by treating financing instruments independent of each other in a multinomial logit model, we isolate the appeal for individual financing instruments.^7^ In addition, we estimate the base model using the classic Heckman selection model (Heckman 1979; Heckman et al. 2006) in which the volume decision depends on the initial decision to raise capital, implying that the decision for the choice of instrument is redundant. Our results remain consistent after applying the three models.

This study contributes to the literature on corporate finance and political economy by offering evidence on how economic policy uncertainty, ownership structure, and governance mechanisms affect financing decisions. The use of a better estimation methodology, namely a three-step sequential framework with a wide range of instruments, is an important contribution of this paper. The model helps to remove sample selection endogeneity concerns. It also helps to establish that the three decisions are not independent and should be analyzed sequentially. Besides improving estimation methodology, this study also contributes to the literature by quantifying the difference between the average volume of financing using either debt or equity securities during the sample period. The role of corporate ownership structure, particularly the presence of institutional investors in the capital raising process, is another pertinent contribution of this paper.

Our findings have implications for investors and policymakers alike. Recent open editorials (Vandevelde 2020; Warsh 2020) highlight, in the context of the Covid-19 pandemic that a loose monetary policy environment and direct intervention by central banks in the secondary markets may induce a moral hazard for issuers and investors. This is particularly relevant to the finding that firms raise more capital during periods of high economic uncertainty. In uncertain situations like the Covid-19 pandemic, the heightened uncertainty increases firms' propensity to borrow through banks, but higher risk aversion and decreased liquidity cause banks to curtail their lending resulting in a sluggish economic recovery.^8^ In response, central banks often loosen monetary policy to encourage lending. The anecdotal evidence suggests that higher demand for debt associated with higher economic uncertainty implies that there is a need for scrutiny of such policies as they may pose a threat to the safety of the financial system through excessive lending and often to failing firms. Therefore, it is pertinent for capital market regulators and financial regulators such as the central bank to assess the behavior of firms raising capital during periods of high uncertainty along with their preferred mode of financing, either debt-based or equity-based, to help shape the monetary policy decisions.

Moreover, the findings are helpful for both corporate investors and shareholders who are seeking to determine the target capital structure of their firms in light of changing economic conditions. For example, institutional shareholders tend to prefer debt over equity when seeking capital. A further increase in leverage during uncertain times will increase bankruptcy costs and affect credit ratings. Therefore, corporate investors such as bond holders may impose leverage rationing as a way to reduce bankruptcy costs to the firm.

The remainder of the paper is organized as follows. The next section presents a brief literature review and develops testable hypotheses. In “Empirical methodology” section presents the empirical methodology used to support this research. In “Variables” section presents the variables used in this study. In “Data sources and descriptive statistics” section describes the data and its sources along with summary statistics. In “Empirical results” section we discuss the results of our empirical analysis; robustness tests are discussed in “Robustness checks” section, and “Conclusions” section concludes the paper.

## Literature review and hypotheses development

Economic policy uncertainty not only affects the profitability of firms but also hampers corporate investment decisions (Baker et al. 2016; Gulen and Ion 2016). Specifically, it affects the decisions to meet their capital requirements (Giambona et al. 2020). The financial flexibility to raise capital using alternative methods, such as bank loans, bonds, and equity has associated costs. Bolton and Freixas (2000) suggest that, depending on the level of information asymmetry, riskier firms prefer bank loans, whereas less risky firms tap the bond markets, and firms in between prefer both equity and bonds.

The empirical literature on the determinants of the choice of the financial instrument remains focused on debt versus equity (Badoer and James 2016; Dong et al. 2012a, b; Jung et al. 1996; MacKie-Mason 1990; Gomes and Phillips 2012); plain vanilla instruments versus hybrid securities (Lewis et al. 2003), or a specific class of instruments such as debt or bank loans (Boubakri and Saffar 2019; Crouzet 2018). However, there is growing evidence suggesting that firms’ choices differ during periods of uncertain economic conditions. On the contrary, studies such as Zeira (1990), Pindyck (1982), and Pindyck and Rubinfeld (1998) found that businesses raise capital less frequently during periods of economic uncertainty. This view is supported by Çolak et al. (2018), who offer evidence of less frequent issuance of debt and equity because of elevated market frictions generated by economic and political uncertainty.

In contrast, several studies suggest that uncertainty raises firms’ capital requirements for investment including internal financing (Atta-Mensah 2004; Klein 1977; Hartman 1972), debt financing as a gap-filling arrangement (Badoer and James 2016), or due to a higher demand for “safe” bonds (Giambona et al. 2020). This leads us to the following hypothesis regarding how economic policy uncertainty affects businesses in their decision to raise capital:

### **Hypothesis 1**

Firms increase financing (both in number of issuances and dollar volume) when economic uncertainty rises and use debt instruments to fulfill this increased demand for capital.

Empirical work related to firms’ policy choices regarding financing differs due to firm-specific attributes such as size, profitability, and growth. These attributes are empirically related to their leverage and ownership structure (Sun et al. 2016; Jensen et al. 1992). Capital structure theories such as pecking order theory (Myers and Majluf 1984), agency cost theory (Jensen and Meckling 1976), signalling theory (Nagar et al. 2019), and static trade-off theory (Leland 1994), suggest that businesses prefer debt to equity when raising external funds due to tax advantages associated with debt, enhanced creditors’ monitoring, and shareholders’ desire for control (Admati et al. 2018; Lemmon and Zender 2019; Crouzet 2018).

Despite the preference for debt, shareholders face difficult choices when firms raise capital. Choosing equity dilutes their ownership stake (Lemmon and Zender 2019; Admati et al. 2018; Boubakri and Ghouma 2010; Ellul 2008; Harris and Raviv 1998), while the use of debt instruments increases bankruptcy costs (Glover 2016; Fama 1980; Masulis 1988). The choice of instrument for raising capital is thus guided by the nexus of shareholders’ desire for control and management of bankruptcy risk. Pecking order theory postulates that a hierarchy of financing instruments exists based on the associated financing costs. While the signalling theory asserts that management uses debt issuance as a mechanism to offer signal to the market about its optimistic future outlook.

Bogle (2018) reports that family/individual shareholdings have significantly declined in the US from 92% in 1945 to 27% in 2018 while at the same time institutional ownership under asset management companies has increased from 8% in 1945 to above 70% in 2018. He et al. (2019) endorse the view that institutional ownership is beneficial to firms because it improves monitoring and consequently reduces agency costs.

Institutional shareholders are primarily concerned with the interests of their clients (Bogle 2018) and may prefer leverage, even though it could be detrimental to the firm’s value (Admati et al. 2018; Boubaker et al. 2017; Ben-Nasr, et al. 2015). However, the assumption that institutional investors are homogenous may lead to a incorrect inference as they may have different motivations and time horizons for investment, leading to different choices regarding capital structure (Elyasiani and Jia 2010; He et al. 2019). There are institutional investors who exert monitoring pressure on management for better long-term performance, while others seek short-term returns, and it is the former that reduces agency costs of debt (Zhang and Zhou 2018). Hence, we divide institutional investors into two groups: long-term and short-term investors. Institutional investors, such as insurance companies, banks, and other corporate shareholders that invest on behalf of their customers, can also influence firms’ financial decisions (Goergen et al. 2019). Since each institutional investor may have different investment objectives, time horizons, and return requirements, we expect the short-term investors to prefer debt which would enable them to gain short-term returns, while the long-term investors to prefer equity to avoid risk in the long run.

Li and Qiu (2021) offer evidence of a decline in debt ratios during periods of high economic policy uncertainty (EPU). However, this analysis does not account for institutional shareholding and its types. We assert that the impact of institutional investors’ categories during periods of economic uncertainty is more pronounced and, consequently, should be analyzed when firms are raising capital and during the selection of securities. This leads to the following two hypotheses regarding how institutional investors influence financing decisions:

### **Hypothesis 2**

Institutional investors with long-term investment objectives prefer to raise capital (both in issuance frequency and issuance volume) using equity instruments.

### **Hypothesis 3**

Institutional investors with short-term investment return expectations prefer debt financing.

## Empirical methodology

There is a vast literature on security issuance providing several explanations for why firms raise capital and their choice of financing instruments (Baker and Wurgler 2002; Myers and Majluf 1984; Jensen and Meckling 1976; Modigliani and Miller 1958). Pecking order theory builds a hierarchical approach to financing, suggesting that firms’ financing decisions follow a unique order: (1) internal resources to avoid external financing costs, (2) debt financing to exploit tax shields, and (3) equity financing (Dong et al. 2012a, b; Khawaja et al. 2019; Shyam-Sunder and Myers 1999). According to static trade-off theory, firms strive to achieve an optimal leverage level by maximizing tax shields associated with debt financing. Furthermore, investors demand a higher premium for equity investment due to higher information asymmetry and greater risk (Myers 1977; Myers and Majluf 1984), leading firms to favor debt financing (Bradley et al. 2016; Nagar et al. 2019; Pástor and Veronesi 2013; Waisman et al. 2015).

The decision to raise capital through a specific financing instrument and the amount thereof are not only directly interrelated but are also indirectly affected by firm-specific and macroeconomic factors. Consequently, the determinants for the decisions to raise capital, the choice of instrument, and volume can differ from each other (Ashraf et al. 2020). Using a system of equations is desirable for such policy decisions that may be applied to a common relationship with real choices.^9^

A simultaneous equation model not only addresses endogeneity concerns due to sample selection bias but also accounts for policy choices at the appropriate level of the decision-making process. We propose that under a sequential framework, the external financing process starts with a binary decision to raise capital followed by the choice of instrument decision and the volume decision.

Since the volume of issuance and the choice of instrument can only be observed for firms that raise capital, this creates sample selection bias (Heckman 1979). In this case, the initial decision of whether to raise funds and the subsequent decision about the choice of instrument posit a double selection bias. To address this bias, we apply the triple selection model based on Heckman et al. (2006) that helps alleviate endogeneity concerns by applying exclusion restrictions at the appropriate steps. Among others, Akashi and Horie (2022), Kehinde et al. (2021), Misman and Bhatti (2020), Ashraf et al. (2020), Brown (2011), Zhang et al. (2015), Wetzels and Zorlu (2003), and Lee (1982) have applied the double selection criteria albeit in different context. We further control for firm- and year-fixed time invariant omitted variable bias to capture unique trends of each issuer and year. We also use robust standard errors to address heteroskedasticity.

**Fig. 1:**
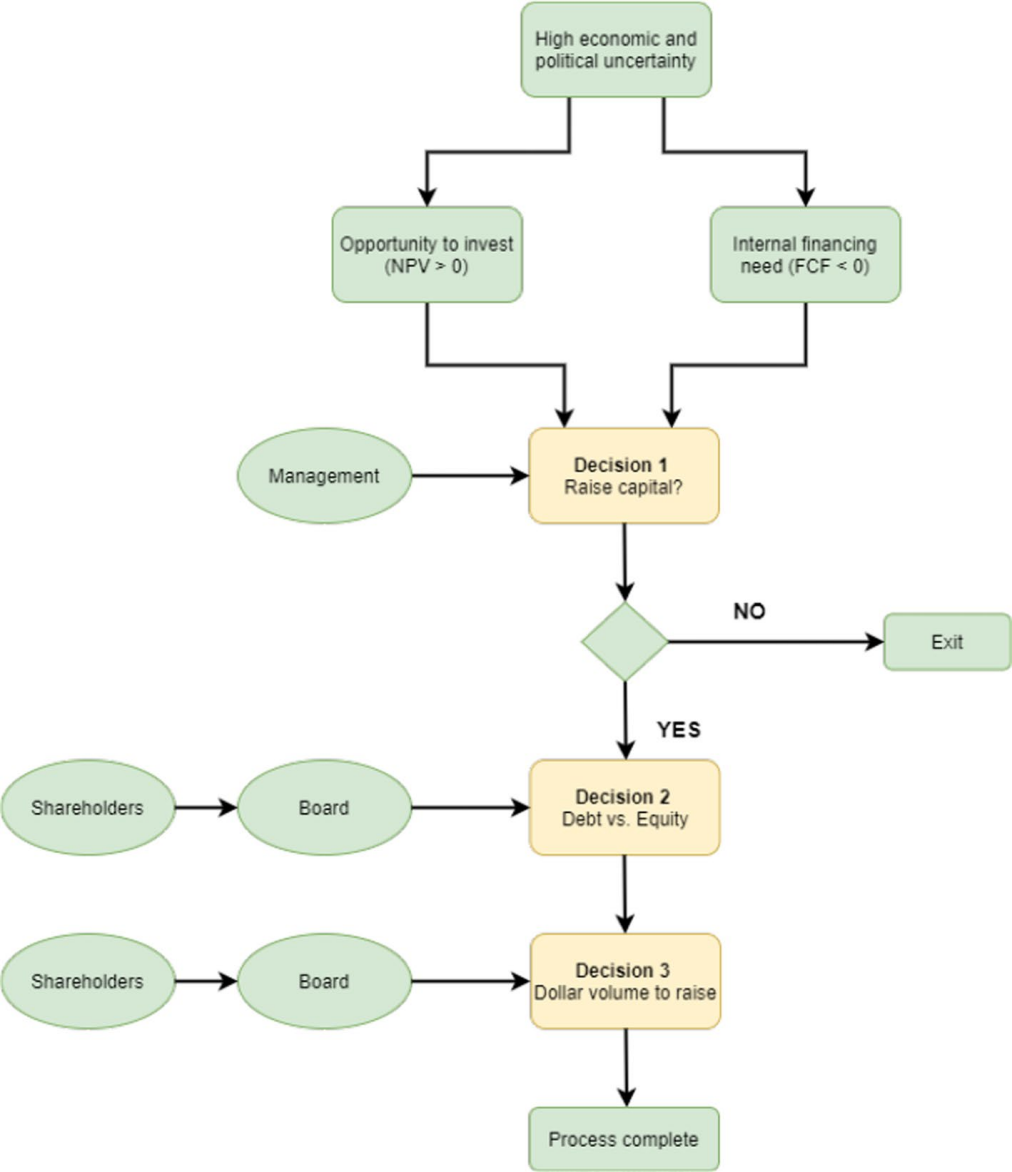
A sequential framework of the decision-making process to raise capital. The figure shows that during periods of uncertainty, firms may come across opportunities to invest in projects with positive Net Present Values (NPV) or require capital because of negative Free Cash Flows (FCF). Shareholders delegate the first decision to exploit management skills (Shibata and Nishihara 2010). Once the decision is made, the subsequent decisions about security choice and dollar volume incorporate shareholder interests represented by the board of directors

The empirical methodology in this study is based on the premise that a firm’s policy decision to raise capital follows a three-step sequential decision process, as shown in Fig. 1. In the basic financing model, once a firm makes a policy decision to raise capital, the firms (i = 1, 2, …, M) choose financial instruments among *J* alternatives based on decreasing levels of desired control and higher risk levels. We can only observe the volume of capital raised and the actual choice j, where $j\in \left\{1, \dots .,J\right\}$, not the decision $I_{j}^{*}$, a latent continuous variable reflecting the desired level of control and relative riskiness.

The sequential decision framework can be developed by following a classical form of the simultaneous equation model as below in (1):

1 $${\text{Issue}}\;{\text{equation:}}\;I_{it}^{*} = {\varvec{X}}_{{{\varvec{it}}}} {\varvec{\gamma}} + \mu_{it}$$

In Eq. (1), $I_{it}^{*}$ is a continuous latent (unobserved) variable, a linear function of explanatory variables, whose value determines the decision of raising capital. The ${\varvec{X}}_{{{\varvec{it}}}}$ is the vector of independent variables and ${\varvec{\gamma}}$ is a vector of unknown parameters. The disturbance term $\mu_{it}$ represents the random element (dependent on the yearly dummy)^10^ in the issue decision where $I_{it}^{*} > 0$ if firm *i* raises capital during year *t*, otherwise firm *i* does not raise capital during year *t*. Since $I_{it}^{*}$ is unobserved and we only observe whether the firm raises capital or not, we estimate the following equation:

2 $${\text{Estimated}}\;{\text{issue}}\;{\text{equation:}}\;\Pr \left( {I_{it} = 1{|}{\varvec{X}}} \right) = {\text{Pr}}(I_{it}^{*} > 0|{\varvec{X}}) = {{\varvec{\Phi}}}\left( {{\varvec{X}}_{{{\varvec{it}}}} \user2{\gamma^{\prime}}} \right)$$

where $I_{it}$ is a dummy variable that is one if the firm raises capital in year $t$ and zero otherwise and ${{\varvec{\Phi}}}$ represents the standard normal cumulative distribution function (cdf), i.e., we use a probit model.

We suppose that firms choose the type of instrument based on a propensity score, denoted as $C_{it}^{*}$, which is a linear function of independent variables ${\varvec{Y}}_{{{\varvec{it}}}}$. That is, we have the following choice equation:

3 $${\text{Choice}}\;{\text{equation:}}\;C_{it}^{*} = {\varvec{Y}}_{{{\varvec{it}}}} {\varvec{\beta}} + \alpha_{1} \lambda_{1,it} + \varepsilon_{it}$$

where ${\lambda }_{1,it}$ is the Heckman (1979) style inverse Mills ratio, from Eq. (2), to deal with the sample selection bias from the first decision and ${\varepsilon }_{it}$ is a random (uncorrelated) disturbance term. Using the propensity score, firms choose the instrument as follows:

4 $${\text{Instrument}}\;{\text{choice:}}\;C_{it} = \left\{ {\begin{array}{*{20}l} {Capital\;not\;raised = 0} \hfill & {\quad if \;C_{it}^{*} \le 0} \hfill \\ {Loan = 1} \hfill & {\quad if \;0 < C_{it}^{*} \le \eta_{1} } \hfill \\ {Bond = 2} \hfill & {\quad if\; \eta_{1} < C_{it}^{*} \le \eta_{2} } \hfill \\ {Convertible \;bond = 3} \hfill & {\quad if \;\eta_{2} < C_{it}^{*} \le \eta_{3} } \hfill \\ {Preferred \;equity = 4} \hfill & {\quad if \;\eta_{3} < C_{it}^{*} \le \eta_{4} } \hfill \\ {Common\; equity = 5} \hfill & {\quad if \;C_{it}^{*} > \eta_{4} } \hfill \\ \end{array} } \right.$$

where $C_{it}$ is an indicator variable representing the firm’s instrument choice and the unknown $\eta$’s satisfy $0 < \eta_{1} < \eta_{2} < \eta_{3} < \eta_{4}$. The instrument choice ($C_{it}$) takes values following the pecking order theory and the order of these categories reflects the preference of shareholders to limit dilution of ownership (Myers and Majluf 1984). Since $C_{it}^{*}$ is an unobserved latent variable, we estimate the relationship in the underlying Eq. (3) with $C_{it}$ as the dependent variable and using ordered probit regression following Chiburis and Lokshin (2007).

Equations (1) and (3) resolve the simultaneity bias in the firm’s decision regarding the choice of instrument to raise funds. However, we are still missing the decision for the volume of capital, defined as ratio of the amount of capital raised to firm assets, that is raised conditional on the choice of instrument. Following Chiburis and Lokshin (2007), we assume that volume of capital raised, $VOL_{ijt}$ for $j \in \left\{ {0,1,2,3,4,5} \right\}$, is a linear function of independent variables ${\varvec{Z}}_{{{\varvec{it}}}}$. In case a firm makes multiple issues by more than one type of security in a year, the choice of instrument is determined by taking the highest volume of capital raised by the firm across all instruments in that year. Then, we have the following:

5 $${\text{Volume}}\;{\text{equation:}}\;VOL_{ijt} = \left\{ {\begin{array}{*{20}l} {{\varvec{Z}}_{{{\varvec{it}}}} \varvec{\varphi }_{0} + \alpha_{2,0} \lambda_{2,it} + \xi_{it,0} } \hfill & {\quad if\; C_{it} = 0} \hfill \\ {{\varvec{Z}}_{{{\varvec{it}}}} \varvec{\varphi }_{1} + \alpha_{2,1} \lambda_{2,it} + \xi_{it,1} } \hfill & {\quad if\; C_{it} = 1} \hfill \\ {{\varvec{Z}}_{{{\varvec{it}}}} \varvec{\varphi }_{2} + \alpha_{2,2} \lambda_{2,it} + \xi_{it,2} } \hfill & {\quad if \;C_{it} = 2} \hfill \\ {{\varvec{Z}}_{{{\varvec{it}}}} \varvec{\varphi }_{3} + \alpha_{2,3} \lambda_{2,it} + \xi_{it,3} } \hfill & {\quad if\; C_{it} = 3} \hfill \\ {{\varvec{Z}}_{{{\varvec{it}}}} \varvec{\varphi }_{4} + \alpha_{2,4} \lambda_{2,it} + \xi_{it,4} } \hfill & {\quad if\; C_{it} = 4} \hfill \\ {{\varvec{Z}}_{{{\varvec{it}}}} \varvec{\varphi }_{5} + \alpha_{2,5} \lambda_{2,it} + \xi_{it,5} } \hfill & {\quad if\; C_{it} = 5} \hfill \\ \end{array} } \right.$$

where $\varphi_{j}$’s are vectors of unknown parameters that vary based on the choice of instrument, $\lambda_{2,it}$ is the inverse Mills ratio from estimation of Eq. (3) using the ordered probit regression, and, for each $j \in \left\{ {0,1,2,3,4,5} \right\}.$ In above equations the disturbance terms $\mu_{it}$, $\tilde{\varepsilon }_{it}$, and $\xi_{it}$ are assumed to be jointly normally distributed with an unknown correlation coefficient between disturbance terms.

The following section discusses the variables for empirical estimations in this study. “Appendix 1” lists the variables along with their definitions.

## Variables

### Economic policy uncertainty

**Fig. 2:**
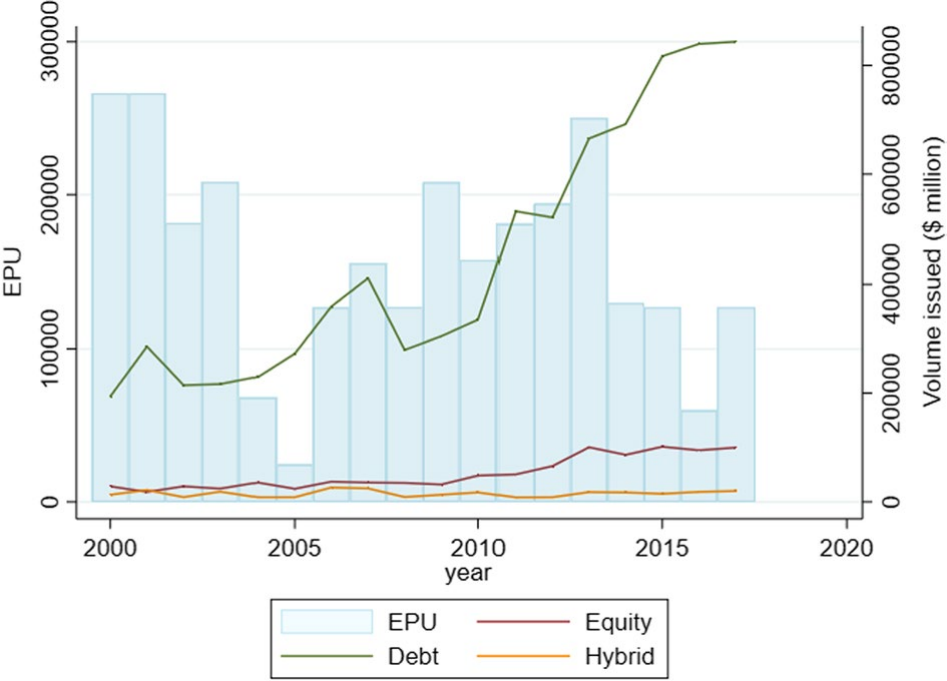
Volume issuance data of sample firms from 2000 to 2017 (2018 is omitted because of incomplete data for that year). The EPU index is scaled to match the issuance trend in volume. The y-axis on the left shows the scaling for the EPU index, while the y-axis on the right shows the dollar volume of capital raised

**Fig. 3:**
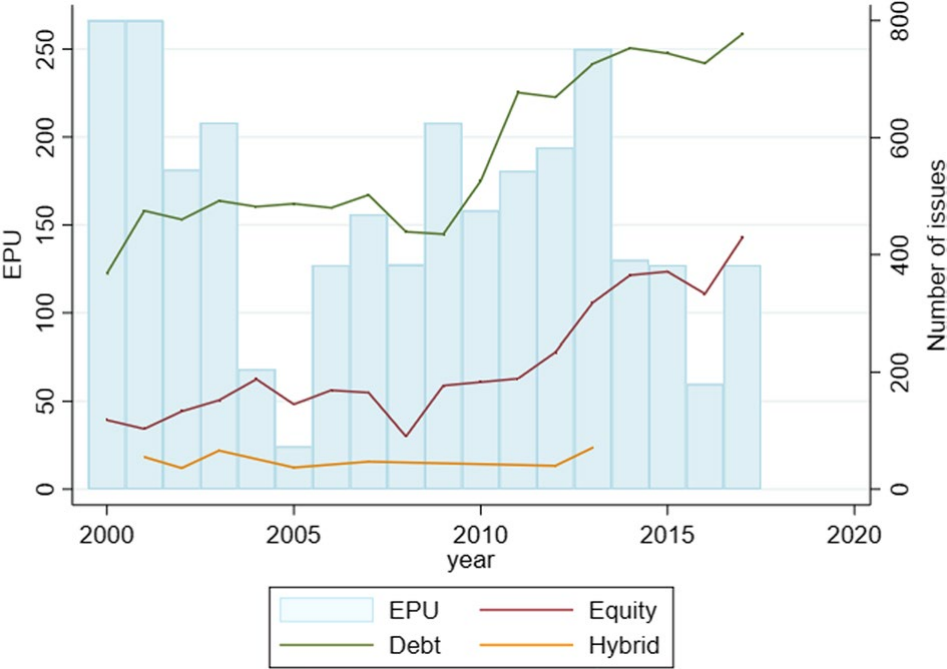
The number of instruments used by the sample firms from 2000 to 2017 (2018 is omitted because of incomplete data for that year). The y-axis on the left shows the scale for the EPU index, while the y-axis on the right shows the number of issues made by a certain instrument

Several studies use the economic policy uncertainty index as a proxy for economic uncertainty.^11^ Instead of using a binary variable for the global financial crisis often used in empirical studies to capture economic policy uncertainty, we use the variable *EPU* as an end-of-year index value from the economic policy uncertainty index developed by Baker et al. (2016) and available in Bloomberg Professional Services.^12^ A higher index value represents a greater magnitude of uncertainty. The movement of the index values over our sample period can be observed in Figs. 2 and 3.

### Ownership structure

#### Ownership concentration: desire for control

Due to the dominance of institutional investors in the US and apparent differences in underlying investment objectives, beneficiaries, and time horizons, we follow Zhang and Zhou (2018) and divide institutional investors into two sub-categories. We term the first category as *Institutional investor*, which includes mutual funds, hedge funds, private equity funds, and venture capital funds. The second category is termed *Long-term investor,* which includes endowment funds, pension funds, sovereign-wealth funds, and financial institutions such as insurance companies, banks, and other corporates.

We use additional categories of shareholders as control variables in empirical estimations. These include government ownership (Boubakri and Saffar 2019; Liu et al. 2011; Li and Zhang 2010; Su 2010; Borisova et al. 2015) and individual /family ownership (Lin et al. 2013). We also include a control variable for concentration of ownership (Holderness 2009; Keasey et al. 2015; Donelli et al. 2013). The variable *Concentration* represents the equity ownership stake of the largest shareholder in the firm.

Since the choice of financing instrument and dollar volume to raise have a pronounced effect on ownership control, we include ownership-related variables in Eqs. (2) and (3) only. This follows from the premise that the initial decision to raise capital is purely technical and is based on the skills and expertise of management (Shibata and Nishihara 2010).

### Other control variables

#### Corporate governance mechanisms

##### CEO duality

It is widely agreed in both theory and practice that the independence of the boards of directors help to reduce agency costs, especially in the absence of monitoring by shareholders. Ideally, a strong and independent board is better positioned to protect the interests of shareholders (Ferreira and Laux 2016).

The agency problem can intensify when such firms raise capital where CEOs have greater control, as is the case where CEO is also the chairperson of the board of directors (Korkeamäki et al. 2017). Since US firms have preferred debt over the past several decades (Graham et al. 2015), CEO duality can be consequential in decisions regarding the choice of financing instrument. This is because CEO duality may increase agency costs, and lower cost efficiency and profitability (Pi and Timme 1993) and hence encourage equity issuance (Jung et al. 1996). However, Brickley et al. (1997) find evidence that CEO duality results in lowering agency costs, and firms can benefit from issuing debt (Jung et al. 1996). We include *CEO duality* as a binary variable to indicate whether the firm’s CEO is also the chairperson of the board.

##### Board size

Board size can affect a firm’s choice of financing instrument. Pearce and Zahra (1992) suggest that firms with a large board size have a greater reliance on debt financing as members of larger boards fail to reach agreement on capital structure decisions (Eisenberg et al. 1998). In contrast, small boards have fewer communication and coordination problems, helping to achieve consistent and timely decisions on capital structure. Berger et al. (1997) find a negative association between board size and leverage. A limitation in this strand of literature is the focus on firm leverage without consideration of the efficiency of the financing process, especially the choice of financing instrument. Prior studies identify that debt is generally preferred to equity (Frank and Shen 2019; Graham et al. 2015; Fama and French 2021); this finding is largely missing in the context of board size. *Board size* is kept as a control variable representing the number of members on the firms’ boards of directors.

##### Golden parachute

Mansi et al. (2016) find a positive relationship between the presence of compensation contracts and cost of debt. They suggest that severance contracts incentivise CEOs to increase firm risk. Chakravarty and Rutherford (2017) concur, noting that protection clauses like golden parachute are associated with higher costs of debt. Cremers et al. (2007) find that golden parachute clauses make debt-based securities more appealing to issuers. Similarly, Wald et al. (2012) report that the presence of golden parachute clause affects the cost of debt financing. Hence, we include the variable *Golden parachute* as a binary variable to show the existence of this clause in firms’ severance contracts.

##### Analyst coverage

Autore and Kovacs (2010) find that higher equity issuance is associated with low information asymmetry. Chang et al. (2006) report that firms covered by fewer analysts are less likely to issue equity as opposed to debt; however, when they do so, it is in larger amounts.

We use two variables to control for the impact of analysts’ opinions on the decision to raise capital. The variable *Analyst coverage* represents the number of analyst recommendations reported for the firm. Furthermore, analyst forecast dispersion represents information asymmetry among analysts and is more pronounced in the subsample with lower earnings quality (Wang et al. 2014). We include the variable *Analyst variance* to reflect the diversity of opinion among analysts represented by the standard deviation in earnings estimates by analysts covering a firm divided by price per share.

##### Insider optimism

Investors keenly watch the trading transactions of insiders such as the CEO, chairperson, key management members, and board members, to assess firm prospects. For example, investors respond more favourably to insider purchases (Goergen et al. 2019) by considering it as a positive signal (Chang and Watson 2015). To reflect how optimistic a firm’s insiders are about the firm’s prospects we generate the variable *Insider optimism* by using the equation below:

4 $$Insider\;optimism = max\left\{ {0,\left( {MVP_{t} - MVS_{t} } \right)/MVP_{t} } \right\}$$

where *MVP*_*t*_ and *MVS*_*t*_ represent the market value of shares bought and sold by insiders during year *t*. The *Insider optimism* variable ranges between 0 and 1, showing an insider’s level of optimism. For the computation of *Insider optimism,* we use the purchase and sale of shares and exclude all other transactions such as vesting of stock options. We expect a positive association of *Insider optimism* with a higher volume of equity issuance.

#### Firm specific and macroeconomic factors

Prior studies suggest that several firm-specific factors help explain a firm’s capital raising decision, including the choice of instrument and dollar volume (Altunbaş et al. 2010; Dong et al. 2012a, b; Lewis et al. 2003). We include variable *Firm Size* (log of total assets) to control for the size of the firm (Altunbaş et al. 2010; Sakai et al. 2010), *Leverage* (debt-to-equity ratio) to account for firm leverage (Berlin and Loeys 1988; Altunbaş et al. 2010), *Profitability* (return-on-assets ratio) to control for profitability (Lemma and Negash 2014), and *Market Optimism* (market-to-book ratio) to control for market’s expectation about firm prospects (Dong et al. 2012a, b).

We further use *Cash* (cash-to-total-assets ratio) and a binary variable, *Free cash flow* that is equal to 1 if the firm has positive free cash flows, 0 otherwise. We expect a negative association of both variables with all capital raising decisions as firms with excess internal resources prefer to avoid increasing leverage or diluting ownership concentration.

To control for macroeconomic conditions, we include *GDP growth,* indicating annual growth in US GDP (Işik et al. 2017), and *Interbank rate*, which is a proxy for the US Federal funds rate (Mendoza 2010; Altunbaş et al. 2010).

## Data sources and descriptive statistics

The sample is comprised of all non-financial US listed firms^13^ on the NYSE, Nasdaq, and AMEX for the period beginning 2000 until 2018. Financial statement data is acquired from Compustat. Data for financial instruments and the volume of capital raised is extracted from the SDC Platinum database. Records of all privately-owned firms are dropped. Issuance data is merged with that of listed firms, including firms with an issuance record, during the sample period resulting in a sample consisting of 2545 issuers and 4289 non-issuers.

Ownership data is acquired from the Thomson Reuters Institutional Ownership database, and corporate governance data from Datastream ASSET4 for the sample firms. Data is merged by matching firm tickers from Compustat. We also locate records in which ownership data is available but missing governance data. The missing data on corporate governance variables are hand-collected from the proxy statements filed with EDGAR. Data on the economic policy uncertainty index and other macroeconomic variables is obtained from Bloomberg Professional Services and the data for insider transactions is extracted from Thomson Reuters Insiders. Finally, the Institutional Broker’s Estimate System (I/B/E/S) database is used to collect data on analyst coverage and dispersion. We remove all records with missing observations of firm assets, debt, and common equity. Further, we drop records with missing information of ownership and governance. Consequently, the final sample is made up of 45,635 firm-year records.

### Descriptive statistics

Figures 2 and 3 depict the relationship between *EPU* and the issuance of capital. The *EPU* index shows a relatively high standard deviation, which is largely because of the spikes in economic uncertainty during the crisis periods of 2000–2001 (dotcom) and 2007–2009 (global financial crisis) observable in Figs. 2 and 3.

Figure 2 shows a comparison of the issuance trend in terms of volume. A major takeaway is that debt has been the major source of capital which is in line with the literature (Myers and Majluf 1984; Admati et al. 2018). Common equity lagged by a wide margin, although in recent years the gap has narrowed. Convertible bonds and preferred equity are not among the major instruments used by firms. Interestingly, the rate of growth in debt is higher, on average, during periods of economic uncertainty.

Figure 3 displays the pattern of issuance classified by the type of instruments. Debt-based instruments continue to be the preferred source for raising capital. The figure shows a trend of frequent security issuance during the crisis years. Years 2000–2001 accompany a sharp rise in issuance frequency. Similarly, years 2008–2013 witness relatively high *EPU* levels accompanied by a consistent rise in security issues, particularly bonds and loans.

Although Figs. 2 and 3 indicate a preference for either equity-like instruments (common equity and preferred equity) or debt-like instruments (loans and bonds), it does not explain the extent to which firms prefer one over the other. We measure this potential difference in issuance volume within debt and equity by applying the Blinder–Oaxaca decomposition procedure (Jann 2008) whereby we divide the volume into two groups, namely ‘equity’ and ‘debt’. The equity group contains common and preferred equity while the debt group includes loans, bonds, and convertible bonds.

The results for the Blinder–Oaxaca decomposition procedure are shown in Table 1. The geometric mean of the volume issuance in a year through debt-based securities amounts to US$584.73 million versus US$488.33 million raised through equity financing indicating a difference of 27.63% on average. The coefficient for difference is significant at the one percent level. Further, adjusting coefficients of equity to the level of debt would lead to a rise in issuance volume in equities by a factor of about 19.73%, while the difference of 7.09% remains unexplained. The adjusted coefficients are shown in “Appendix 2”. The results follow from Figs. 2 and 3 regarding the general preference for debt.

**Table 1:** Blinder–Oaxaca decomposition of equity and debt issuance applied to a sample of 6834 publicly listed US firms over the sample period starting 2000 until 2018

*Volume*	Overall	Adjusted
Debt	584.73*** (1.8897)	584.73*** (1.8897)
Equity	488.33*** (4.8059)	458.16*** (23.598)
Difference	1.1974*** (0.0124)	1.2763** (0.0659)
Explained		1.1973*** (0.0164)
Unexplained		1.0709*** (0.0461)
Observations		9726

Dependent variable *volume* is the logarithm of the dollar volume of capital raised. The coefficients are generated after retransforming them into the original scale of millions of US dollars. The row ‘explained’ indicates the proportion of increase in equity to the level of debt issuance that would be generated by an adjustment in the list of determinants shown in “Appendix 2”. Probability of coefficient estimates from the model greater than standard statistics are provided in parentheses with ***p < 0.01, **p < 0.05, *p < 0.1***. Parentheses contain robust standard error estimates. Asterisks correspond to the outcome of the z-test from the model

Table 2 reports the descriptive statistics for non-dummy variables in three panels for all firms: issuers, non-issuers, and the differences in means between issuers and non-issuers. Among the ownership structure variables, it is evident that institutional investors (such as asset management companies and fund managers) form a single dominant group of shareholders who hold, on average, 83 percent of the overall shareholdings among the sample firms.

**Table 2:** Descriptive Statistics of non-dummy variables representing public US firms over the sample period 2000 until 2018

	All-firms			Panel A: issuers		Panel B: non-issuers		Panel C: difference
Variable	Obs	Mean	SD	Obs	Mean	Obs	Mean	
EPU	45,635	154.456	63.245					
Institutional Investor	39,780	82.922	21.737	29,060	83.982	10,720	80.051	3.931***
Long-term investor	39,780	8.463	14.251	29,060	8.382	10,720	8.682	0.300*
Individual	39,780	5.696	16.506	29,060	5.018	10,720	7.536	− 2.518***
Government	39,780	0.014	0.885	29,060	0.005	10,720	0.038	− 0.033***
Concentration	39,780	87.020	12.658	29,060	87.061	10,720	86.911	− 0.150
Insider optimism	45,635	0.152	0.358	32,228	0.181	13,407	0.083	0.098***
Market optimism	38,748	3.188	6.404	29,687	3.295	9061	2.837	0.458***
Board size	30,386	8.789	3.242	24,125	8.704	6261	9.117	− 0.413***
Firm size	45,617	6.397	2.428	32,223	6.601	13,394	5.904	0.697***
Analyst coverage	31,686	9.310	7.892	25,231	10.057	6455	6.388	3.669***
Analyst variance	31,686	0.704	0.378	25,231	0.727	6455	0.617	− 0.109***
Leverage	45,452	0.189	0.210	32,111	0.206	13,341	0.147	0.059***
Cash	45,149	0.163	0.199	31,875	0.153	13,274	0.185	− 0.032***
Profitability	45,354	− 0.076	0.398	32,140	− 0.069	13,214	− 0.092	0.023***
GDP growth	45,635	1.961	1.429					
Interest rate	45,635	1.619	1.943					

Panel A shows the summary statistics of variables for firms that have raised capital during the sample period. Panel B shows the statistics of variables for firms that did not raise any capital during the sample period. Panel C shows the mean differences of issuer and non-issuer characteristic variables with significance levels ***p < 0.01, **p < 0.05, *p < 0.1. Mean difference analysis for macroeconomic variables does not apply to individual firms and, consequently, are not presented. Probability of coefficient estimates from the model greater than standard statistics are provided in parentheses. Parentheses contain robust standard error estimates. Asterisks correspond to the outcome of the z-test from the model

There are some notable differences between issuers and non-issuers. The difference-in-means analysis suggests that in each of the ownership categories, issuers are statistically different from non-issuers at the one percent significance level. Issuers are more likely to have higher institutional ownership as compared to non-issuers. Insider optimism is more pronounced amongst issuers, who are not only larger in size but also more leveraged. Greater insider optimism for issuers suggest higher growth potential as compared to non-issuers that are more liquid with larger boards of directors. From the difference-in-means analysis, we can assert that large firms with higher institutional ownership are more likely to raise capital due to lower information asymmetry, better economies of scale, and better access to the capital market.

Table 3 reports the correlation matrix with coefficients representing correlations across major independent (non-binary) variables used in this study. Generally, the correlation coefficients are in line with our expectations. Factors that can be adversely affected by the economic uncertainty on a stand-alone basis include firm’s profitability, size, and institutional investor ownership. Among the covariates that elevate during periods of economic uncertainty is *Insider optimism* that suggests a signalling mechanism.

**Table 3:** Correlation matrix with coefficients representing correlations across major independent (non-binary) variables used in the study

	EPU	Long-term investor	Institutional investor	Concentration	Insider optimism	Market optimism	Board size	Firm size	Analyst coverage	Analyst variance
EPU	1									
Long-term investor	0.0704	1								
Institutional investor	− 0.0388	− 0.102	1							
Concentration	− 0.0416	− 0.154	0.7958	1						
Insider optimism	0.0259	− 0.0468	− 0.0034	− 0.0037	1					
Market optimism	− 0.0118	− 0.0178	0.017	0.0038	0.0496	1				
Board size	0.0221	0.1846	− 0.0048	− 0.0369	− 0.0454	− 0.0189	1			
Firm size	− 0.0217	0.279	− 0.0193	− 0.0705	− 0.1061	− 0.0419	0.478	1		
Analyst coverage	− 0.0147	0.1962	0.0014	− 0.0481	− 0.0185	0.0965	0.226	0.6355	1	
Analyst variance	− 0.0431	0.0738	0.0498	0.0142	0.0091	0.0325	0.0607	0.1868	0.2463	1

The sample includes annual macroeconomic data and annual firm-related data of 6834 publicly listed firms in the US. Sample period starts 2000 until 2018

## Empirical results

Before we proceed to discuss the empirical results, it is pertinent to investigate whether the model is appropriate to perform the sequential analysis. Our model has several independent variables and there is a possibility of multicollinearity in our sample. To check for the presence of multicollinearity, we measure the Variance Inflation Factor (VIF). Since ownership variables are not part of the first equation, we account for variables on economic policy uncertainty, governance mechanisms, information asymmetry, and other firm-specific control variables. Table 4 shows that all VIF estimates are less than 3 and most are less than 2, suggesting the absence of multicollinearity across the regressors (O’Brien 2007).

**Table 4:** Variance Inflation Factor (VIF) measure for multicollinearity

Variable	VIF
Firm size	2.89
Analyst coverage	1.91
Cash	1.39
Board size	1.33
Leverage	1.23
Profitability	1.19
EPU	1.11
Interest rate	1.11
Golden parachute	1.09
GDP growth rate	1.08
Market optimism	1.05
Insider optimism	1.03
CEO duality	1.02
Board attendance	1.02
Mean VIF	1.3

The table includes variables on governance, information asymmetry, and firm-specific factors. The sample selection model does not include firm ownership variables in the first equation on capital issuance and, consequently, are excluded from VIF analysis

Table 5 reports the empirical results based on the simultaneous decision framework developed in “Empirical methodology” section. Before presenting the estimation results, it is pertinent to investigate whether the adoption of the sample selection framework is appropriate for empirical analysis. A Wald test with null hypothesis that the disturbance terms in the *Issue* and *Choice* equations and *Issue* and *Volume* equations are uncorrelated *(H*_0_: *ρ* = 0*)* is reported at the bottom of Table 5. We observe a positive estimate for *ρ* indicating that unobservable variables affecting the issuance decision tend to occur with those affecting the choice decision. Although there is some difference between the size of these tests, together they indicate the presence of endogenous sample selection bias and support the use of a sample selection model. The residuals in the *Volume* equation are found to be heteroscedastic, so all statistical inference is based on robust standard errors. The empirical estimations are presented after controlling for the year-fixed effects and firm-fixed effects. However, the results are reported only for the variables of interest.

**Table 5:** Empirical estimations based on the Heckman three-stage ordered probit model with firm-fixed effects and year-fixed effects and robust standard errors

Variables	Issue	Choice	Volume
EPU	0.0010*** (0.0002)	− 0.0183*** (0.0053)	− 0.0006*** (0.0002)
Long-term investor		− 0.0354*** (0.0077)	− 0.0042 (0.0065)
Institutional investor		− 0.0056** (0.0027)	− 0.0106* (0.0058)
Individual		0.0032 (0.0031)	− 0.0035 (0.0052)
Government		− 0.0530 (0.2710)	− 0.1566* (0.0941)
Concentration	0.0008 (0.0010)	0.0040 (0.0035)	− 0.0002 (0.0078)
Golden parachute	0.0379 (0.0321)	0.3079*** (0.0558)	− 0.1421*** (0.0310)
CEO duality	0.0024 (0.0183)	0.0222 (0.0419)	− 0.0579*** (0.0213)
Insider optimism	0.0653*** (0.0221)	0.1073*** (0.0375)	− 0.1221*** (0.0320)
Market optimism	0.0056*** (0.0016)	0.0063*** (0.0024)	− 0.0006 (0.0043)
Board size	0.0250*** (0.0036)	0.0053 (0.0126)	0.0244*** (0.0044)
Analyst coverage	− 0.0072*** (0.0015)	0.0060 (0.0042)	0.0233*** (0.0030)
Analyst variance	0.0382 (0.0294)	− 0.1092* (0.0616)	− 0.3229*** (0.0553)
Firm size	0.2431*** (0.0092)	− 0.0396 (0.0462)	− 0.5863*** (0.0451)
Leverage	0.8245*** (0.0539)	0.4202*** (0.1423)	− 0.4395*** (0.1199)
Cash	0.0435 (0.0796)	1.2362*** (0.2257)	2.1627*** (0.2119)
Free cash flow	− 0.0841*** (0.0259)		
Profitability	− 0.8910*** (0.0805)		
Interest rate	0.0077 (0.0049)	0.0302 (0.0623)	0.0003 (0.0057)
GDP Growth rate	0.0736*** (0.0066)	1.5626*** (0.5085)	− 0.0406*** (0.0120)
Constant	− 2.6434*** (0.1208)	6.6998*** (0.8157)	
*Ρ*			− 0.6268*** (0.1594)
Selectivity bias			− 0.1081** (0.0530)
Firm—fixed effects	Yes	Yes	Yes
Year—fixed effects	Yes	Yes	Yes
Wald test of indep. eqns. (ρ = 0) χ^2^(1)			15.47***
Observations	20,976	20,976	20,969

The sample includes data from 6834 publicly listed firms in the US. The sample period is from 2000 until 2018. Firms’ decisions follow the sequence shown in Fig. 1. The first decision on issuance is represented by the binary dependent variable *Issue*. The *Choice* category variable is in the second column takes up values following pecking order theory as follows: Loan = 1; Bond = 2; Convertible bond = 3; Preferred equity = 4; Common equity = 5. The selectivity bias variable indicates the presence of sample selection bias. *Ρ* indicates the correlation between error terms in output and participation equations. Probability of coefficient estimates from the model greater than standard statistics are provided in parentheses with ***p < 0.01, **p < 0.05, *p < 0.1***. Parentheses contain robust standard error estimates. Asterisks correspond to the outcome of the z-test from the model. Year fixed-effects and firm fixed-effects are included; however, the estimated coefficients are not reported (Long tables are available upon request). Variable definitions are given in “Appendix 1”

Table 5 reports the empirical results in three panels: *Issue*, *Choice*, and *Volume,* representing the sequential decisions to raise capital. The *Issue* panel reports the results for Eq. (1), the *Choice* panel reports the results for Eq. (2), and the *Volume* panel reports the estimation results for Eq. (3). Since the order for the choice of instruments is based on pecking order theory (Loan = 1; Bond = 2; Convertible bond = 3; Preferred equity = 4; Common equity = 5), coefficients in the *Choice* equation with positive signs imply a tendency towards common and preferred equity, while a negative coefficient reflects an inclination towards debt instruments such as loans and bonds.

From Table 5, it is evident that *EPU* plays a significant role in the initial decision to raise capital. The coefficient of *EPU* is positive and significant in the *Issue* equation, suggesting that firms raise capital more frequently during periods of higher economic uncertainty. This is in line with the findings of Atta-Mensah (2004) and Abel (1983) suggesting that uncertainty increases the demand for capital.

Table 5 also reports that, conditional upon the issuance decision, firms prefer to choose debt instruments as suggested by the negative and significant coefficient in the *Choice* equation. This can be attributed to higher market uncertainty leading to higher premium requirements from investors for raising equity capital (Pástor and Veronesi 2013). This result supports the finding of Nagar et al. (2019) that uncertainty leads to greater information asymmetry, and that higher uncertainty leads to debt financing.

As indicated by Table 5, the negative and significant coefficient of *EPU* in the *Volume* equation suggests that an appetite for debt financing does not lead to higher issuance volume. This suggests that firms do not prefer to exacerbate financial risk through leverage during periods of higher economic uncertainty. Hence, we find partial support for our first hypothesis that firms are more likely to increase financing (in number but not in volume) by using debt instruments during periods of economic uncertainty.

Regarding Hypotheses 2 and 3, we find negative and significant coefficients for both categories of institutional investors in both equations, suggesting that firms with a higher proportion of institutional ownership are more likely to raise capital through debt financing and, conditional on the choice decision, in lower volumes. The relationship highlights the risk-averse nature of these investors whereby the sample firms simultaneously attempt to keep a check on ownership dilution while curtailing financial risk. This is in line with Bogle (2018) who suggests that institutional ownership plays an active role in firms’ decision-making.

The inclination towards debt as the source of capital, albeit in lower volumes, provides support for the ownership control hypothesis whereby shareholders prefer debt over equity to avoid ownership dilution (Lemmon and Zender 2019; Boubakri and Ghouma 2010; Ellul 2008). These findings also support Admati et al. (2018) and Boubaker et al. (2017) that institutional investors prefer debt and make slower adjustments to capital structure, as suggested by the negative sign in the *Volume* equation. Among the other ownership variables, *Concentration* is statistically insignificant in all three equations. Hence, there is insufficient evidence to suggest that a rise in concentration of shareholder ownership affects the decision-making process at any stage.

Regarding governance mechanisms, we do not find a significant influence of the concentration of power on the *Issue* and *Choice* decisions—suggested by the insignificant coefficients of *CEO duality,* while the *Volume* decision has a negative coefficient. This indicates that firms with CEO duality do not consistently follow a pattern for raising capital. This contradicts the findings of Korkeamäki et al. (2017) that CEOs with dual roles enhance their control by increasing leverage and complements the findings of Jensen (1993) that boards find it difficult to perform their functions in the presence of CEO duality.

The insignificant coefficient of *Golden parachute* in the *Issue* equation, and positive and significant coefficient in the *Choice* equation, suggest that the presence of a golden parachute clause does not affect the *Issue* decision. However, when such firms decide to raise capital, equity financing is preferred. These results contradict the findings of Mansi et al. (2016) and Chakravarty and Rutherford (2017) that severance contracts incentivise firms to make risky decisions.

The coefficients of *Insider optimism* and *Market optimism* are positive and significant in the *Issue* and *Choice* equations. This signals insiders’ faith in the stability and growth of the firm. These findings are in line with market timing theory that firms prefer to raise capital when there is an optimism for growth (Baker and Wurgler 2002). In addition, the former has a negative relationship with the *Volume* decision. We infer that optimistic insiders hold on to their control and avoid large issues, leading to ownership dilution.

Among the variables on information asymmetry, the negative and significant coefficient of *Analyst coverage* in the *Issue* equation suggests that firms covered by a greater number of analysts tend to raise capital less frequently. Results of firm-specific control variables are also in line with our expectations. We do not discuss them here for brevity.

Overall, we find the empirical evidence to support for Hypothesis 1 indicating that firms raise capital more often and prefer debt instruments, albeit not in greater volume. Further, there was no evidence to support Hypothesis 2, that long-term institutional investors prefer equity financing. Finally, the empirical results support Hypothesis 3 that short-term institutional investors prefer debt-based instruments.

## Robustness checks

In this section we conduct additional tests to support the empirical findings that are presented in the previous section.

### Political uncertainty

The empirical evidence thus far supports the notion that at firm level the decision to raise capital is affected by economic policy uncertainty. An efficient way to measure policy uncertainty, besides the use of conventional indices provided by Baker et al. (2016), is by analyzing political uncertainty. Political uncertainty is likely to rise in the US when the executive and legislative bodies of the government are controlled by separate political parties, a phenomenon termed as ‘divided government’. This is because a divided government has historically failed to generate important legislation because of the President having opposing views than the legislature (Edwards et al. 1997; Rogers 2005). Hence, the expectations of businesses and their executives in terms of legislative outcome are barely met under a divided government, leading to uncertainty. The partisan differences between Democrats and Republicans are one of the key factors for political uncertainty in the US (Waisman et al. 2015).

To account for political uncertainty, we use the interaction of variables *EPU* and political uncertainty (PU). *PU* is a dummy variable equal to unity if the President is from a party different than the majority party in the House. To control for the impact of firm size, we add another interaction variable of *EPU* and *Size*. By introducing these two interaction variables, we control for the impact of firm size and political uncertainty in the sequential decision framework.

Table 6 reports the empirical results after incorporating both interaction variables. Interestingly, the coefficient of the interaction term *EPU* × *SIZE* is insignificant in the three equations, implying that the decision for financing among large firms is not associated with higher economic uncertainty. However, political uncertainty coupled with economic policy uncertainty affects the issuance decision and the subsequent choice decision, as reflected by the significant coefficient of *EPU* × *PU.* Together, these findings suggest that firms prefer to raise capital during periods of political uncertainty coupled with economic uncertainty by using debt instruments. This is in line with our previous finding that the choice of debt instruments during periods of higher policy uncertainty is related to information asymmetry, leading to higher premium requirements from investors when they raise equity capital (Pástor and Veronesi 2013). This finding implies that firms faced with political uncertainty, coupled with economic policy uncertainty, prefer to use internal financing (if available). However, this finding should be interpreted with caution because it is plausible that political divergence may not fully reflect the behaviour of firms towards political risk.

**Table 6:** Empirical estimations based on the Heckman three-stage ordered probit model with firm-fixed effects and year-fixed effects and robust standard errors

Variables	Issue	Choice	Volume
EPU	0.0002 (0.0007)	− 0.0192*** (0.0053)	0.0015 (0.0020)
EPU × SIZE	0.0001 (0.0001)	0.0001 (0.0001)	− 0.0003 (0.0002)
EPU × PU	0.0002* (0.0001)	− 0.0076*** (0.0028)	0.0001 (0.0002)
Long-term investor		− 0.0356*** (0.0077)	− 0.0037 (0.0066)
Institutional investor		− 0.0056** (0.0027)	− 0.0105* (0.0058)
Individual		0.0032 (0.0031)	− 0.0035 (0.0052)
Government		− 0.0521 (0.2699)	− 0.1590* (0.0944)
Concentration	0.0008 (0.0010)	0.0040 (0.0035)	− 0.0003 (0.0078)
Golden parachute	0.0298 (0.0325)	0.3098*** (0.0559)	− 0.1500*** (0.0316)
CEO duality	0.0037 (0.0183)	0.0252 (0.0421)	− 0.0625*** (0.0215)
Insider optimism	0.0657*** (0.0221)	0.1079*** (0.0375)	− 0.1238*** (0.0319)
Market optimism	0.0056*** (0.0016)	0.0063*** (0.0024)	− 0.0007 (0.0042)
Board size	0.0251*** (0.0036)	0.0051 (0.0126)	0.0246*** (0.0045)
Analyst coverage	− 0.0072*** (0.0015)	0.0060 (0.0042)	0.0232*** (0.0031)
Analyst variance	0.0358 (0.0294)	− 0.1086* (0.0615)	− 0.3235*** (0.0552)
Firm size	0.2288*** (0.0162)	− 0.0556 (0.0545)	− 0.5448*** (0.0572)
Leverage	0.8280*** (0.0540)	0.4202*** (0.1423)	− 0.4397*** (0.1204)
Cash	0.0390 (0.0796)	1.2405*** (0.2258)	2.1575*** (0.2112)
Free cash flow	− 0.0826*** (0.0259)		
Profitability	− 0.8927*** (0.0806)		
Interest rate	0.0118** (0.0053)	0.0309 (0.0624)	0.0010 (0.0065)
GDP Growth rate	0.0710*** (0.0068)	1.5717*** (0.5066)	− 0.0419*** (0.0119)
Constant	− 2.5211*** (0.1561)	6.3812*** (0.8735)	
*Ρ*			− 0.6288*** (0.1589)
Selectivity bias			− 0.0963* (0.0493)
Firm—fixed effects	Yes	Yes	Yes
Year—fixed effects	Yes	Yes	Yes
Wald test of indep. eqns. (ρ = 0) χ^2^(1)			15.67***
Observations	20,976	20,976	20,969

The sample includes data from 6834 publicly listed firms in the US. The sample period is from 2000 until 2018. Firms’ decisions follow the sequence shown in Fig. 1. The first decision on issuance is represented by the binary dependent variable *Issue*. The *Choice* categorical variable in the second column takes up values following pecking order theory as follows: Loan = 1; Bond = 2; Convertible bond = 3; Preferred equity = 4; Common equity = 5. The selectivity bias variable indicates the presence of sample selection bias. *Ρ* indicates the correlation between error terms in output and participation equations. Probability of coefficient estimates from the model greater than standard statistics are provided in parentheses with ***p < 0.01, **p < 0.05, *p < 0.1***. Parentheses contain robust standard error estimates. Asterisks correspond to the outcome of the z-test from the model. Year fixed-effects and firm fixed-effects are included; however, the estimated coefficients are not reported (Long tables are available at request). Variable definitions are given in “Appendix 1”

Regarding the results for other variables, we do not see a major shift in the results except for the level of significance of the *EPU* variable in the *Issue* equation. Qualitatively, there is no major deviation from previous findings.

### Relaxing the strict categorical order: multinomial logit model

The strict ordered categorical variable in the *Choice* model based on the pecking order theory assumes that firms select instruments to raise capital in a specific order. However, it is likely that a firm’s choice of instrument for raising capital is not strictly ordered and it may choose the instrument based on the economic policy environment, ownership structure, or their financial condition. Furthermore, one may argue that the issuance volume is the first decision it subsequently determines which security (debt or equity) to choose depending on whether the required amount will exceed the firm’s debt capacity.

As a robustness check, we use a variable for the choice of instrument that does not follow a specific order. Essentially, by removing the order we witness every instrument’s appeal to the firm given other independent variables. We achieve this by applying the multinomial logit model as presented by Dubin and McFadden (1984) and Bourguignon et al. (2007) with sample selection in the *Choice* equation.

Table 7 reports the estimation results based on a multinomial logit model in the *Choice* equation. There is no major difference in the empirical findings for the *Issue* and *Choice* equations as we observe a greater tendency to raise capital with a preference for loans and bonds under political and economic uncertainty. Through the *Volume* equation, we infer that there is a general trend of lower issuance volume, except in large firms. In addition, we find that long-term institutional investors avoid equity financing, which supports previous findings.

**Table 7:** Empirical estimation based on a multinomial logit model for the *Choice* equation

Variables	Issue	Choice					Volume
		Loan	Bonds	Convertible bonds	Preferred equity	Common equity	
EPU	0.0002 (0.0007)	0.0054 (0.0127)	0.0076 (0.0128)	− 0.0090 (0.0132)	0.0198 (0.0159)	0.0021 (0.0127)	0.0020 (0.0018)
EPU × SIZE	0.0001 (0.0001)	− 0.0004 (0.0013)	− 0.0004 (0.0013)	0.0016 (0.0014)	− 0.0029 (0.0018)	− 0.0003 (0.0013)	− 0.0011*** (0.0003)
EPU × PU	0.0002** (0.0001)	0.0067** (0.0028)	0.0049* (0.0028)	0.0025 (0.0029)	0.0029 (0.0034)	0.0043 (0.0028)	0.0025*** (0.0003)
Long-term investor		0.0571 (0.0403)	0.0646 (0.0399)	0.0121 (0.0442)	− 0.0065 (0.0668)	− 0.1218*** (0.0425)	− 0.0738*** (0.0115)
Institutional investor		0.0230 (0.0290)	0.0188 (0.0289)	0.0416 (0.0326)	0.0524 (0.0583)	0.0192 (0.0291)	− 0.0044 (0.0039)
Individual		− 0.0052 (0.0380)	− 0.0117 (0.0380)	− 0.0191 (0.0393)	− 0.0104 (0.0491)	− 0.0052 (0.0379)	0.0094** (0.0039)
Government		− 0.3194 (9.9629)	− 1.2884 (9.9822)	− 14.0662 (969.15)	− 15.8856 (3443.01)	− 0.0986 (9.9539)	1.7348*** (0.2869)
Concentration	0.0008 (0.0010)	− 0.0400 (0.0400)	− 0.0419 (0.0399)	− 0.0593 (0.0432)	− 0.0623 (0.0679)	− 0.0418 (0.0400)	0.0076 (0.0057)
Golden parachute	0.0297 (0.0330)	0.1946 (0.6371)	0.0655 (0.6356)	0.2378 (0.6652)	0.3660 (0.8790)	− 0.0357 (0.6449)	0.0658** (0.0278)
CEO duality	0.0036 (0.0183)	0.1703 (0.4432)	0.2058 (0.4431)	0.0459 (0.4516)	− 0.0779 (0.5203)	− 0.2217 (0.4443)	− 0.2653*** (0.0336)
Insider optimism	0.0655*** (0.0222)	− 0.1975 (0.5772)	− 0.2029 (0.5774)	0.0504 (0.5848)	− 0.4025 (0.6689)	− 0.1189 (0.5779)	0.0402 (0.0329)
Market optimism	0.0056*** (0.0015)	0.0167 (0.0210)	0.0224 (0.0210)	0.0184 (0.0213)	− 0.0206 (0.0304)	0.0116 (0.0208)	− 0.0134*** (0.0034)
Board size	0.0251*** (0.0035)	0.1872** (0.0916)	0.2040** (0.0916)	0.0188 (0.0928)	0.0386 (0.1068)	0.0853 (0.0918)	− 0.0867*** (0.0069)
Analyst coverage	− 0.0072*** (0.0015)	− 0.0010 (0.0315)	0.0186 (0.0314)	0.0768** (0.0322)	− 0.0107 (0.0423)	0.0685** (0.0317)	0.0140** (0.0057)
Analyst variance	0.0358 (0.0297)	0.3051 (0.8403)	0.4623 (0.8421)	0.0685 (0.8534)	0.1052 (0.9297)	− 0.1047 (0.8396)	− 0.5530*** (0.0609)
Firm size	0.2288*** (0.0163)	1.1497*** (0.2767)	1.3221*** (0.2807)	− 0.2229 (0.2879)	0.3870 (0.3711)	− 0.2588 (0.2756)	− 1.3918*** (0.0977)
Leverage	0.8285*** (0.0515)	7.4758*** (0.8705)	8.8602*** (0.8924)	7.3853*** (0.9022)	3.6881*** (1.1532)	4.8665*** (0.8390)	− 3.6041*** (0.4564)
Cash	0.0393 (0.0757)	− 2.3805 (1.7130)	− 1.3334 (1.7265)	2.2095 (1.7153)	0.9291 (1.9151)	0.6766 (1.6982)	− 0.5516** (0.2787)
Free cash flow	− 0.0803*** (0.0240)						
Profitability	− 0.8934*** (0.0634)						
Interest rate	0.0117** (0.0053)	0.2808** (0.1222)	0.2431** (0.1222)	0.2828** (0.1247)	0.2925** (0.1427)	0.2030* (0.1225)	0.0305*** (0.0065)
GDP Growth rate	0.0709*** (0.0068)	0.7634*** (0.1790)	0.6134*** (0.1790)	0.4675** (0.1818)	0.3451* (0.2034)	0.4817*** (0.1792)	0.1360*** (0.0170)
Constant	− 2.5231*** (0.1562)	− 26.5117*** (4.3367)	− 27.8902*** (4.4142)	− 12.6266*** (4.4205)	− 14.4169*** (5.3824)	− 8.2984* (4.2568)	19.0410*** (1.3393)
*Ρ*							− 0.2339** (0.0943)
Selectivity bias (Eq. 2)		33.4769*** (1.6244)	31.8580*** (1.6480)	27.8274*** (1.6338)	24.7157*** (1.7285)	27.3773*** (1.5961)	
Selectivity bias (Eq. 3)		7.2401*** (0.3815)	− 5.6055*** (0.8186)	1.8063** (0.7456)	− 3.0184* (1.7204)	5.9013*** (0.6259)	
Firm—fixed effects	Yes	Yes	Yes				
Year—fixed effects	Yes	Yes	Yes				
Wald test of indep. eqns. (ρ = 0) χ^2^(1)							6.15**
Observations	20,976	20,994	20,994	20,994	20,994	20,994	20,968

The model includes firm-fixed effects and year-fixed effects and robust standard errors. The sample includes data from 6834 publicly listed firms in the US. The sample period is from 2000 until 2018. Firms’ decisions follow the sequence shown in Fig. 1. The first decision on issuance is represented by the binary dependent variable *Issue*. The *Choice* columns indicates a firm’s choice of instrument without any order. The selectivity bias variables indicate the presence of sample selection bias. The selectivity bias (Eq. 3) estimates are for separate variables for each *Choice* category but are shown in a single row for brevity. *Ρ* indicates the correlation between error terms in output and participation equations. Probability of coefficient estimates from the model greater than standard statistics are provided in parentheses with ***p < 0.01, **p < 0.05, *p < 0.1***. Parentheses contain robust standard error estimates. Asterisks correspond to the outcome of the z-test from the model. Year fixed-effects and firm fixed-effects are included; however, the estimated coefficients are not reported (Long tables are available upon request). Variable definitions are given in “Appendix 1”

### Heckman selection model

The underlying hypothesis with the above empirical estimation is that firms are concerned with shareholders’ desire for control and/or financial stability in their *Choice* decision. However, if a firm’s decision to raise capital is unaffected by the choice of instrument, it still presents a sample selection problem after controlling for firm-fixed effects for the time-invariant factors. To test for the robustness of our results, we adopt the classic Heckman Sample Selection model (Heckman 1979; Heckman et al. 2006). By adopting this model, we incorporate only the *Issue* and *Volume* equations after controlling for sample selection bias and applying the exclusion restriction.

Table 8 reports the results based on the Heckman Selection model. The empirical findings are generally in line with the main models for the *Issue* and *Volume* decisions in Tables 4 and 5. A slight exception is the negative effect of uncertainty coupled with firm size on the *Issue* decision. However, the coefficient is very small and significant at the 10 percent level.

**Table 8:** Empirical estimation based on the Heckman two-stage model without the *Choice* equation with firm-fixed effects and year-fixed effects and robust standard errors

Variables	Issue	Volume
EPU	0.0007 (0.0034)	0.0029 (0.0018)
EPU × SIZE	− 0.0002* (0.0001)	− 0.0004** (0.0002)
EPU × PU	0.0005 (0.0018)	0.0003*** (0.0001)
Long-term investor		0.0009 (0.0074)
Institutional investor		− 0.0062* (0.0038)
Individual		− 0.0008 (0.0046)
Government		− 0.0782 (0.0804)
Concentration	− 0.0010 (0.0015)	0.0027 (0.0054)
Golden parachute	0.0428 (0.0539)	0.1224*** (0.0286)
CEO duality	− 0.0509 (0.0387)	0.0392** (0.0196)
Insider optimism	0.0159 (0.0271)	− 0.0278 (0.0224)
Market optimism	0.0040* (0.0021)	0.0003 (0.0030)
Board size	0.0071 (0.0120)	0.0375*** (0.0069)
Analyst coverage	− 0.0077** (0.0032)	0.0183*** (0.0035)
Analyst variance	− 0.0534 (0.0377)	− 0.1992*** (0.0424)
Firm size	0.3412*** (0.0327)	− 0.6048*** (0.0639)
Leverage	1.1334*** (0.1018)	0.1367 (0.1204)
Cash	0.5953*** (0.1303)	0.6622*** (0.2175)
Free cash flow	− 0.0834** (0.0341)	
Profitability	− 0.1835* (0.1019)	
Interest rate	0.0534 (0.0445)	− 0.0037 (0.0042)
GDP Growth rate	0.1178 (0.3211)	− 0.0109* (0.0058)
Constant	− 2.8323*** (0.5228)	5.0286*** (0.6763)
Selectivity bias		0.1853*** (0.0467)
Firm—fixed effects	Yes	Yes
Year—fixed effects	Yes	Yes
Wald χ^2^(21)		710***
Observations	18,307	9504

The sample includes data from 6834 publicly listed firms in the US. The sample period is from 2000 until 2018. Firms’ decisions follow the sequence shown in Fig. 1. The first decision on issuance is represented by the binary dependent variable *Issue*. The selectivity bias variable indicates the presence of sample selection bias. Probability of coefficient estimates from the model greater than standard statistics are provided in parentheses with ***p < 0.01, **p < 0.05, *p < 0.1***. Parentheses contain robust standard error estimates. Asterisks correspond to the outcome of the z-test from the model. Year fixed-effects and firm fixed-effects are included; however, the estimated coefficients are not reported (Long tables are available upon request). Variable definitions are given in “Appendix 1”

### Implied volatility index to measure uncertainty

As an alternate to the economic policy uncertainty index, we use the implied volatility index (VIX) to understand if firms’ capital-raising behaviour is significantly different during uncertain market conditions. Table 9 reports the results with VIX variable replacing *EPU* variable. There are exceptions from the previous findings in the *Issue* and *Volume* decisions as the VIX coefficient is insignificant. However, we observe a continuation of the trend that firms prefer to raise capital using debt financing, as the coefficient of *Choice* decision is negative and significant. We can attribute the deviation in findings in the *Issue* and *Volume* equations to the fact that the stock market is relatively more volatile than *EPU* (Liu and Zhang 2015). Hence, businesses do not respond to changes in market volatility for raising capital more frequently. For the same reason, the decision about *Volume* is not significantly affected.

**Table 9:** Empirical estimations based on the Heckman three-stage ordered probit model with firm-fixed effects and year-fixed effects and robust standard errors

Variables	Issue	Choice	Volume
VIX	− 0.0008 (0.0016)	− 0.0337*** (0.0090)	− 0.0001 (0.0020)
Long-term investor		− 0.0354*** (0.0077)	− 0.0045 (0.0065)
Institutional investor		− 0.0056** (0.0027)	− 0.0106* (0.0058)
Individual		0.0032 (0.0031)	− 0.0035 (0.0053)
Government		− 0.0529 (0.2704)	− 0.1550 (0.0945)
Concentration	0.0006 (0.0010)	0.0040 (0.0035)	− 0.0001 (0.0078)
Golden parachute	0.0288 (0.0320)	0.3082*** (0.0558)	− 0.1362*** (0.0303)
CEO duality	0.0000 (0.0183)	0.0224 (0.0419)	− 0.0561*** (0.0213)
Insider optimism	0.0692*** (0.0221)	0.1070*** (0.0375)	− 0.1238*** (0.0325)
Market optimism	0.0055*** (0.0016)	0.0063*** (0.0024)	− 0.0006 (0.0043)
Board size	0.0256*** (0.0036)	0.0052 (0.0126)	0.0240*** (0.0045)
Analyst coverage	− 0.0068*** (0.0015)	0.0060 (0.0042)	0.0231*** (0.0030)
Analyst variance	0.0280 (0.0293)	− 0.1088* (0.0616)	− 0.3173*** (0.0549)
Firm size	0.2405*** (0.0092)	− 0.0402 (0.0446)	− 0.5850*** (0.0451)
Leverage	0.8188*** (0.0539)	0.4177*** (0.1419)	− 0.4343*** (0.1200)
Cash	0.0399 (0.0795)	1.2364*** (0.2255)	2.1638*** (0.2118)
Free cash flow	− 0.0854*** (0.0258)		
Profitability	− 0.8748*** (0.0798)		
Interest rate	0.0150*** (0.0054)	0.2865*** (0.0625)	− 0.0035 (0.0064)
GDP Growth rate	0.0611*** (0.0072)	− 0.2130** (0.1039)	− 0.0340*** (0.0112)
Constant	− 2.4087*** (0.1208)	6.5781*** (0.7877)	
*Ρ*			− 0.6248*** (0.1608)
Selectivity bias			− 0.0451** (0.0207)
Firm—fixed effects	Yes	Yes	Yes
Year—fixed effects	Yes	Yes	Yes
Wald test of indep. eqns. (ρ = 0) χ^2^(1)			15.10***
Observations	20,976	20,976	20,969

Economic uncertainty is measured by the implied volatility index (VIX). The sample includes data from 6834 publicly listed firms in the US. The sample period is from 2000 until 2018. Firms’ decisions follow the sequence shown in Fig. 1. The first decision on issuance is represented by the binary dependent variable *Issue*. The *Choice* categorical variable in the second column takes up values following pecking order theory as follows: Loan = 1; Bond = 2; Convertible bond = 3; Preferred equity = 4; Common equity = 5. The selectivity bias variable indicates the presence of sample selection bias. *Ρ* indicates the correlation between error terms in output and participation equations. Probability of coefficient estimates from the model greater than standard statistics are provided in parentheses with ***p < 0.01, **p < 0.05, *p < 0.1***. Parentheses contain robust standard error estimates. Asterisks correspond to the outcome of the z-test from the model. Year fixed-effects and firm fixed-effects are included; however, the estimated coefficients are not reported (Long tables are available upon request). Variable definitions are given in “Appendix 1”

## Conclusions

In this paper, we investigate how economic uncertainty drives three decisions in firms’ capital-raising process: the decision to raise capital, the decision about the choice of financing instrument, and the decision about the issuance volume. Instead of analyzing the three decisions separately, we apply a sequential three-step decision-making framework through a simultaneous equation model.

Our findings suggest that during times of high economic uncertainty, firms raise capital more frequently, choose debt-based instruments, and raise higher volumes of capital. When economic uncertainty is coupled with political uncertainty, larger firms abstain from raising capital in higher volumes.

The proportion of ownership by long-term institutional investors (including endowment funds, pension funds, and sovereign-wealth funds) as well as asset management firms (such as hedge funds, advisory firms, private equity, and venture capital) is positively associated with the issuance of debt in lower volumes. In addition, high insider optimism is associated with greater instances of raising capital; this follows a preference for equity financing to raise capital.

Our finding that higher instances of raising capital are associated with high economic uncertainty implies that the appetite for capital increases during such periods. The preference for debt instruments for raising capital supports capital structure theories, including the pecking order theory, the agency cost theory, the signalling theory, and the static trade-off theory. Each suggests that debt is the preferred means of raising capital under different scenarios, including information asymmetry and tax benefits. The role of insider optimism aligns with that of market optimism and endorses market timing theory. This paper also establishes the significant roles of ownership structure and governance mechanisms in the sequential decision-making process of raising capital.

The findings of this paper have certain policy implications. First, the demand for debt instruments during periods of political and economic uncertainty (such as the current Covid-19 pandemic, the dot-com crisis, and the global financial crisis) may threaten the financial system's safety. The response in the form of loose monetary policy and/or direct intervention by central banks in the secondary markets may induce increased borrowing by firms either due to a higher need for working capital or hoarding cash to create a safety cushion.

We acknowledge that the study has a few limitations. Our sample contains only US data; hence, the findings may not be generalized to other markets. Further, given the limitations in acquiring private firm data, our results only depict the trends in public firms. In addition, despite using a range of financial instruments, a broader set of securities (such as notes, warrants, debentures, etc.) could enhance the understanding of firm behavior.

Research on firms’ capital raising behaviour during the Covid-19 pandemic can shed further light on the increase in capital demand during economic crises. Furthermore, additional research can highlight the role of economic uncertainty and insider optimism in other corporate decisions, such as mergers and acquisitions, executive compensation, and project finance. Another potential research avenue is security issuance covering financial management and risk/return analysis from both the firm and the investor perspective.

## Data Availability

We use data from various financial data vendors, and the data is easily accessible from those data vendors. All vendors are listed within the text of the manuscript.

## Appendix 1: Variable definitions

**Table:**

Variable	Definition		Source
Issue	Binary variable which takes the value of 1 if the firm raises capital, 0 otherwise		SDC Platinum
Choice	Categorical variable assigned a value based on the firm’s choice of security. Following are the possible choices: Loan = 1; Bond = 2; Convertible bond = 3; Preferred equity = 4; Common equity = 5		SDC Platinum
Volume	Ratio of dollar volume of capital raised by the firm with total assets		SDC Platinum
EPU	End-of-year index value of the Economic Policy Uncertainty Index	Bradley et al. (2016)	Bloomberg
Concentration	Percentage of ownership by the highest shareholder in the firm	Keasey et al. (2015)	Thomson Reuters
Institutional investor	Percentage of ownership by institutional investors and include mutual funds, hedge funds, advisors, private equity, and venture capital firms	Zhang and Zhou (2018)	Thomson Reuters
Long-term investor	Percentage of ownership in the firm by long-term institutional investors. These include endowments, pension funds, sovereign-wealth funds, and banks	Zhang and Zhou (2018)	Thomson Reuters Ownership
Individual	Percentage of ownership in the firm by individuals and families	Lin et al. (2013)	Thomson Reuters
Government	Percentage of ownership in the firm held by the government	Boubakri and Saffar (2019)	Thomson Reuters
Golden parachute	Binary variable equals to 1, if the firm has a golden parachute or other restrictive clauses, 0 otherwise	Cremers et al. (2007)	Datastream
Board size	Number of members on the board of directors	Eisenberg et al. (1998)	Datastream
CEO duality	Binary variable which takes the value of 1 if the CEO is also the chairperson of the board, 0 otherwise	Korkeamäki et al. (2017)	Datastream
Insider optimism	Level of optimism of a firm insider, calculated as: Max (0, $\frac{{{\text{volume purchased }}{-}{\text{ volume sold}}}}{{\text{volume purchased}}}$)	Goergen et al. (2019)	Thomson Reuters Insiders
Market optimism	Market-to-book value	Dong et al. (2012a, b); Hovakimian (2001)	Compustat
Analyst coverage	Number of analyst recommendations for the firm	Derrien et al. (2016)	I/B/E/S
Analyst variance	Standard deviation in earnings estimates by analysts covering a firm divided by price per share	Derrien et al. (2016)	I/B/E/S
Firm size	Log of total assets of the firm	Autore and Kovacs (2010)	Compustat
Profitability	Return-on-assets	Lemma and Negash (2014)	Compustat
Leverage	Debt-to-assets ratio	Faulkender et al. (2012)	Compustat
Cash	Cash-to-asset ratio	Lewis et al. (2003)	Compustat
Free cash flow	Binary variable equal to 1 if the firm has positive cash flows, 0 otherwise	Lewis et al. (2003)	Compustat
GDP growth	Percentage change in annual GDP	Altunbaş et al. (2010)	Bloomberg
Interbank rate	End-of-year Federal Funds rate	Altunbaş et al. (2010)	Bloomberg

## Appendix 2: Blinder–Oaxaca model

Empirical estimation results from the Blinder–Oaxaca model breaking down the geometric mean difference between equity and debt issuance volume. The column titled, ‘Explained’ shows the adjustment in coefficients that explain a rise of equity issuance volume to the level of debt. The’Unexplained’ column shows the unexplained coefficients. Probability of coefficient estimates from the model greater than standard statistics are provided in parentheses with ****p* < 0.01, ***p* < 0.05, **p* < 0.1***. Parentheses contain robust standard error estimates. Asterics correspond to the outcome of the z-test from the model.

**Table:**

Variables	Explained	Unexplained
EPU	− 0.0008 (0.0012)	− 0.0084 (0.0908)
EPU × SIZE	0.0053 (0.0183)	0.0222 (0.1028)
EPU × PU	− 0.0002 (0.0007)	− 0.0131 (0.0083)
Concentration	− 0.0024** (0.0012)	− 0.2224* (0.1141)
Long-term investor	− 0.0115** (0.0047)	0.0302** (0.0135)
Institutional investor	− 0.0006 (0.0007)	0.1264 (0.0934)
Individual	0.0017 (0.0023)	0.0010 (0.0023)
Government	− 0.0000 (0.0001)	0.0001 (0.0003)
Golden parachute	− 0.0002 (0.0020)	0.0018 (0.0330)
CEO duality	− 0.0036 (0.0023)	0.0181* (0.0101)
Insider optimism	− 0.0032* (0.0017)	− 0.0083** (0.0040)
Market optimism	− 0.0025** (0.0012)	− 0.0055 (0.0045)
Board size	− 0.0016 (0.0052)	0.0008 (0.0365)
Firm size	0.1922*** (0.0228)	− 0.2803** (0.1290)
Analyst coverage	0.0098* (0.0054)	− 0.0063 (0.0226)
Analyst variance	0.0026 (0.0017)	− 0.0228 (0.0260)
Leverage	0.0027* (0.0015)	0.0046 (0.0155)
Cash	− 0.0150*** (0.0051)	− 0.0036 (0.0047)
Interest rate	0.0027** (0.0013)	− 0.0131 (0.0092)
GDP Growth rate	− 0.0000 (0.0004)	− 0.0151 (0.0147)
Constant		0.4621** (0.1871)
Observations	9726	9726

## Abbreviations

3SLS: Three-stage least square
CEO: Chief Executive Officer
EPU: Economic policy uncertainty
OLS: Ordinary least squares
VIX: Implied volatility index

## Footnotes

1 The Economist: Companies have raised more capital in 2020 than ever before. https://www.economist.com/business/2020/12/09/companies-have-raised-more-capital-in-2020-than-ever-before

2 See for example, pecking order theory, signalling theory, and static trade-off theory provide the basis for the preference of debt over equity financing. In contrast, market timing theory advocates semi-strong market efficiency—implying that the decision to raise capital using equity is contingent upon the expectation that stock prices are deemed overvalued (Dong et al. 2012a, b; Stein 1996). A discussion on capital structure theories is provided in “Literature review and hypotheses development” section.

3 For example, the Global Financial Crisis of 2008 or the European Debt Crisis of 2010, and/or the ongoing Covid-19 pandemic crisis.

4 Ownership structure of firms has changed considerably in the past few decades. For example, institutional ownership has increased from 8% in 1945 to above 70% in 2018 among US firms while family/individual shareholdings have significantly declined from 92% in 1945 to 27% in 2018 (Bogle 2018).

5 We note that firms may raise capital in a different order in certain circumstances. For example, firms under financial distress would have the volume decision preceding the choice of instrument. To account for exceptional circumstances, we test for firms’ financing behaviour by removing the strict hierarchical order for the choice of financing instrument as a robustness check.

6 We use data from the US market for two reasons: (1) it witnessed periods of economic growth and recessions during our sample period, making it possible to witness varying levels of EPU; (2) political uncertainty in the US has fluctuated in the past few decades allowing us to use alternative measures for economic uncertainty.

7 This is the property of independence of irrelevant alternatives which means the probability of choosing one option is independent of the other options in the set of choices available to the decision-maker. Recent studies use FinTech and information technology (see for example Kou et al. 2021, 2022).

8 As noted by Acharya and Steffen (2020), Halling et al. (2020), Li et al. (2020), Coakley et al. (2021), Nguyen et al. (2021), Wen et al. (2019) and Bertoni and Groh (2022). To see how model is generalized via double selection issues, the readers are directed to the papers by Akashi and Hories (2022), Kehinde et al. (2021), Lee (1982), Krishnan (1990), Wetzels and Zorlu (2003) and Brown (2011) among others.

9 Most studies that investigate the determinants of financing volume generally assume an absence of relationship between the choice of instrument and the volume decision and thus model them separately. These studies model the relationship as a single equation model such as Ordinary Least Squares (OLS) regression for volume or an ordered probit model for the choice of instruments (Zhang et al. 2015; Zhang and Zhou 2018; Suchard and Singh 2006; Lewis et al. 2003; Jung et al. 1996; Van-de-Ven and Van Pragg 1981). Both models suffer from sample selection bias as they ignore the initial decision for raising capital. Recently, Boubakri and Saffar (2019) modelled the two decisions of issuance and volume using the Heckman Two Stage model. However, they ignore the intermediate decision regarding the choice of security.

10 We refer to Smithson and Merkle (2013) in which we can manipulate disturbance term $\mu_{it}$ in terms of $\mu_{it} = \left( {I_{it}^{*} - \alpha^{\prime}{\varvec{X}}_{{{\varvec{it}}}} } \right)$.

11 See for example Işık et al. (2020) and Li and Qiu (2021).

12 See Alvarado et al. (2021) as an example of use of indices to measure macroeconomic factors.

13 Since our focus is on non-financial firms, firms belonging to the financial sector with SIC codes in the range 6000–6799 are removed from the sample.
